# Associations of [^18^F]PI-2620 Binding with Memory and Phosphorylated Tau 217 in Cognitively Unimpaired Older Adults

**DOI:** 10.2967/jnumed.125.271927

**Published:** 2026-10

**Authors:** Anne Maass, Berta Garcia-Garcia, Niklas Behrenbruch, Beate Schumann-Werner, Eóin N. Molloy, Svenja Schwarck, Michael Rullmann, Anne Hochkeppler, Larissa Fischer, Anna-Therese Büchel, Jose Bernal, Niklas Vockert, Enise I. Incesoy, Wenzel Glanz, Michaela Butryn, Peter Schulze, Kathrin Baldauf, Andrew W. Stephens, Andreas Schildan, Marianne Patt, Gusalija Behnisch, Barbara Morgado, Hermann Esselmann, Constanze I. Seidenbecher, Björn H. Schott, Jens Wiltfang, Henryk Barthel, Osama Sabri, Michael C. Kreissl, Emrah Düzel

**Affiliations:** 1 German Center for Neurodegenerative Diseases, Magdeburg, Germany;; 2 Faculty of Natural Sciences, Otto-von-Guericke-University Magdeburg, Magdeburg, Germany;; 3 Center for Behavioral Brain Sciences, Magdeburg, Germany;; 4 Division of Nuclear Medicine, Department of Radiology and Nuclear Medicine, Faculty of Medicine, Otto-von-Guericke-University Magdeburg, Magdeburg, Germany;; 5 Institute of Cognitive Neurology and Dementia Research, Otto von Guericke University Magdeburg, Magdeburg, Germany;; 6 Department of Nuclear Medicine, University Hospital Leipzig, Leipzig, Germany;; 7 Institute for Neuroscience and Cardiovascular Research, Row Fogo Centre for Research into Ageing and the Brain, Department of Neuroimaging Sciences, University of Edinburgh, Edinburgh, Scotland;; 8 Department Artificial Intelligence in Biomedical Engineering, Friedrich-Alexander-Universität Erlangen-Nürnberg, Erlangen, Germany;; 9 Life Molecular Imaging GmbH, Berlin, Germany;; 10 Section Radiopharmacy, Department of Nuclear Medicine, University Hospital Augsburg, Augsburg, Germany;; 11 Leibniz Institute for Neurobiology, Magdeburg, Germany;; 12 Department for Psychiatry and Psychotherapy, University Medical Center Göttingen, Göttingen, Germany;; 13 German Center for Neurodegenerative Diseases, Göttingen, Germany;; 14 Neurosciences and Signaling Group, Institute of Biomedicine, Department of Medical Sciences, University of Aveiro, Aveiro, Portugal; and; 15 Department of Nuclear Medicine, Municipal Hospital Dessau, Dessau-Roßlau, Germany

**Keywords:** tau PET, PI-2620, Alzheimer disease, episodic memory, phospho-tau 217

## Abstract

[^18^F]PI-2620 is a second-generation tau PET tracer that may detect early tau accumulation in aging. We investigated whether temporal lobe [^18^F]PI-2620 binding is associated with age, sex, genetic Alzheimer disease (AD) risk, plasma biomarkers of AD (plasma phosphorylated tau 217 [p-tau_217_], Aβ_1–42_/Aβ_1–40_), astrogliosis (glial fibrillary acidic protein), and domain-specific cognition in cognitively unimpaired (CU) older adults. **Methods:** In this study, 166 CU older adults (mean age, 72 ± 7 y; females, 46%; apolipoprotein ϵ4 [APOE4] carriers, 23%) and 13 young adults underwent extensive cognitive testing, blood sampling, MRI, and dynamic [^18^F]PI-2620 PET (0–60 min postinjection). Associations of regional [^18^F]PI-2620 distribution volume ratio (DVR) with age, sex, APOE4 genotype, and plasma biomarkers were examined using region-of-interest and voxelwise analyses. Additional imaging markers of age-related pathology included hippocampal volume, medial temporal lobe thickness, white matter (WM) hyperintensities, perivascular spaces, and hippocampal perfusion (R1-derived maps). Associations among temporal lobe DVR, other imaging markers, and domain-specific cognitive performance (from factor analysis) were tested. **Results:** In older adults, temporal [^18^F]PI-2620 binding was higher in women (β = 0.543, *P* < 0.001) and APOE4 carriers (β = 0.395, *P* = 0.030) and was positively associated with plasma p-tau_217_ (β = 0.22, *P* = 0.008). Voxelwise analyses showed age-related increases in basal ganglia signal, whereas WM signal was higher in younger adults. In a multiple regression model, higher temporal DVR (β = −0.36, *P* = 0.003) and lower hippocampal volume (β = 0.22, *P* = 0.007) predicted worse episodic memory and, together with demographic factors, explained approximately 30% of the variance. **Conclusion:** Temporal [^18^F]PI-2620 binding is associated with genetic AD risk, plasma p-tau_217_, female sex, and episodic memory deficits in CU older adults, supporting its sensitivity to early tau pathology, while highlighting the need to consider potential WM binding.

## Visual Abstract

**Figure:**
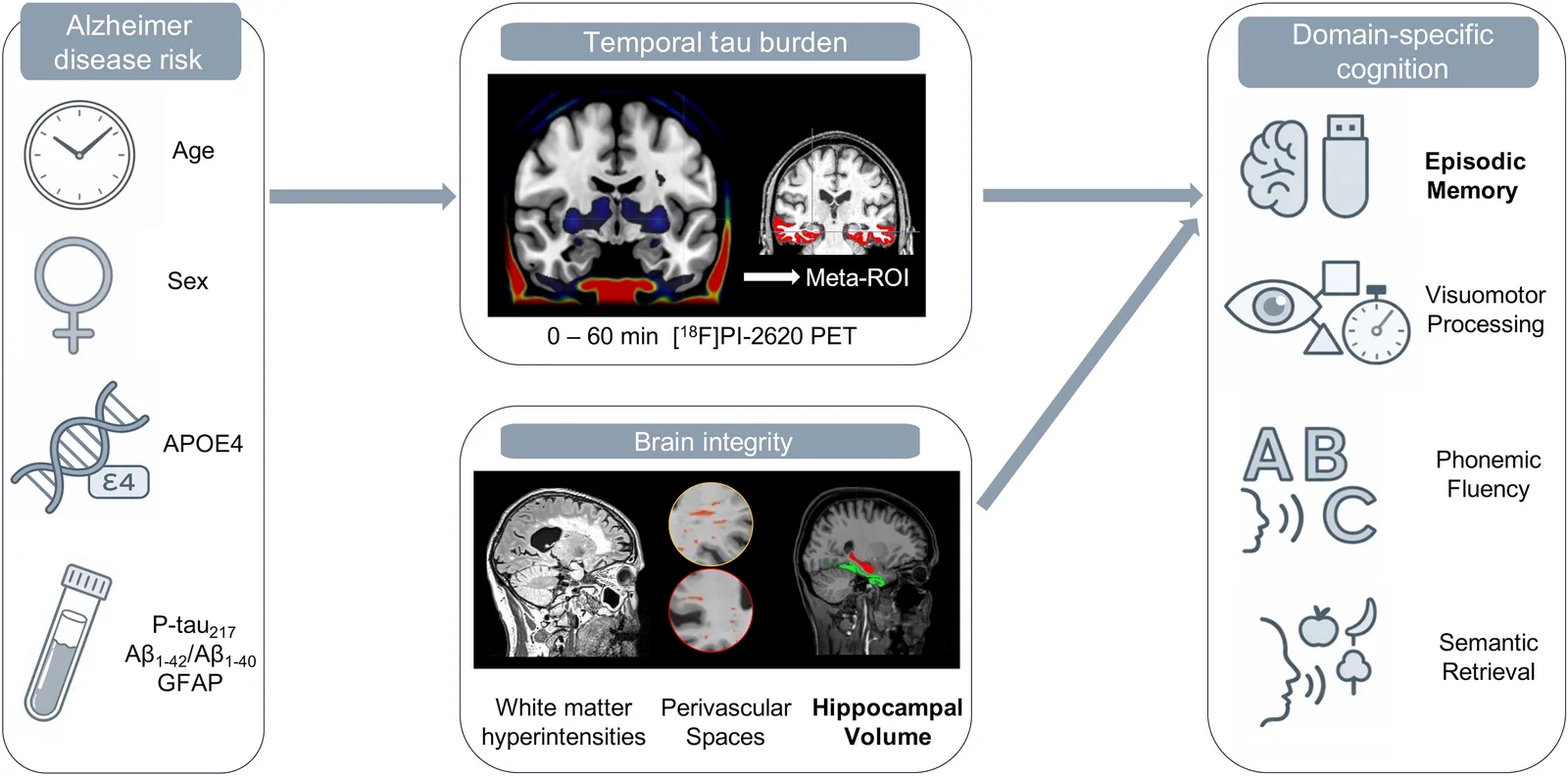

Tau PET enables in vivo assessment of tau pathology in Alzheimer disease (AD) and other tauopathies. Postmortem studies show that tau tangles in the medial temporal lobe (MTL) are common in older adults (1) and may contribute to age-related episodic memory decline.

Among the second-generation tau PET tracers, [^18^F]PI-2620 has demonstrated high affinity for tau isoforms found in AD and non-AD tauopathies and low off-target binding to monoamine oxidases (2–4). In patients with mild cognitive impairment and dementia [^18^F]PI-2620 binding was related to elevated amyloid-β peptide (Aβ) burden and cognitive impairment (5–8). As with other tau tracers, such as flortaucipir (9), several studies reported some degree of off-target binding for [^18^F]PI-2620 in substantia nigra, basal ganglia, choroid plexus, cerebellar vermis, retina, soft tissue, and venous sinuses (2,7,10) and variable white matter (WM) binding has been observed (11).

To date, few studies have investigated how [^18^F]PI-2620 is associated with memory performance or AD-related biomarkers in cognitively unimpaired (CU) older adults (12,13). In 57 CU participants, a higher entorhinal SUV ratio (SUVR) was related to worse memory and language function and to higher phosphorylated tau (p-tau) 181 levels in cerebrospinal fluid. Moreover, no studies have assessed [^18^F]PI-2620 binding patterns in young adults or in very old age (e.g., age over 80 y), when MTL tau pathology is likely expected (1,14). For first-generation tau tracers (e.g., flortaucipir), higher temporal binding has been linked to worse memory in CU older adults (15–17). Overall, it remains debated whether early tangle accumulation in the temporal lobe, which is common in aging, can be detected with the tau tracers currently available (18), such as [^18^F]PI-2620. In addition to AD pathology, other neurodegenerative pathologies and cerebrovascular conditions contribute to cognitive decline in old age, as revealed by postmortem data (14). However, only a few multimodal studies have determined the contributions of various age-related pathologies to cognition in CU older adults in vivo (19,20).

This study aimed to examine the binding patterns of [^18^F]PI-2620 in CU adults and their associations with age, sex, and apolipoprotein ϵ4 (APOE4) genotype; the relationship between temporal tau PET burden and plasma biomarkers; and the associations of tau PET and other age-related brain markers with domain-specific cognition.

## MATERIALS AND METHODS

### Participants

Participants were drawn from the central “Z03” cohort of the Collaborative Research Center SFB1436 (21). Community-dwelling CU adults 60 y or older were recruited in and around Magdeburg, Germany. Inclusion criteria comprised unimpaired cognition and the absence of neurologic or psychiatric disease, major medical illness, diabetes, cognition-affecting medication use, and substance use disorder. Additional MRI and PET eligibility criteria are provided in the supplemental materials, available at http://jnm.snmjournals.org. The study protocol (German Clinical Trials Register: DRKS00032449) was approved by the local ethics committee of the Medical Faculty, Otto-von-Guericke University Magdeburg (200/19) and conducted in accordance with the Declaration of Helsinki. All participants provided written informed consent. By September 2025, 372 participants had completed cognitive testing. Tau PET was performed for 170 eligible older adults and 13 young adults (recruitment ongoing).

### Interview and Cognitive Testing

During visit 1, participants completed the Consortium to Establish a Registry for Alzheimer Disease Neuropsychological Assessment Battery to ensure unimpaired cognitive status. Participants were excluded if their performance in the assessment battery was not normal for age, sex, and education or if their Mini-Mental State Examination score was less than 26. During visit 2, participants received further comprehensive cognitive testing across memory, executive, visuomotor, and language domains (Supplemental Methods).

### Exploratory Factor Analysis on Cognitive Data

Exploratory factor analysis was conducted on cognitive data from 370 participants with assessments at both visits, after exclusion of 1 participant with more than 20% missing data. For each test, the primary performance metric was selected, and tests showing ceiling effects or heterogeneous constructs were excluded. The 18 included cognitive scores were residualized for investigator effects, imputed, and standardized using *z*-scores. Factor analysis was performed using principal axis factoring with oblimin rotation (supplemental materials).

### Assessment of Plasma Biomarkers and APOE4 Genotype

Plasma p-tau_217_, Aβ_1–40_, Aβ_1–42_, and glial fibrillary acidic protein (GFAP) were analyzed using the plasma immunoreaction cartridges on the fully automated Lumipulse G600II system (Fujirebio Diagnostics). We computed an Aβ–tau (AT) term (Aβ_1–40_/Aβ_1–42_ × p-tau_217_), with higher values indicating more pathology. All plasma markers (Aβ_1–40_/Aβ_1–42_ after inversion) were log-transformed to reduce skewness (Supplemental Methods).

### MRI Acquisition and Processing

3-T MRI (Skyra; Siemens Healthineers) was performed and included a 0.8-mm isotropic multiecho magnetization-prepared rapid gradient-echo sequence (MPRAGE) and a 1.0-mm isotropic fluid-attenuated inversion recovery. In addition, a 1-mm isotropic MPRAGE acquired during PET/MRI was used for PET preprocessing. T1-weighted images were processed using FreeSurfer version 7.1 and the Desikan–Killiany Atlas. Bilateral hippocampal volumes and mean MTL cortical thickness (entorhinal, perirhinal, and fusiform gyri) were derived from MR images. Hippocampal volumes were residualized for total intracranial volume, estimated using FreeSurfer’s SAMSEG-based segmentation (21).

Perivascular spaces (PVS) in the centrum semiovale and basal ganglia were segmented from T1-weighted MRI and fluid-attenuated inversion recovery images using a validated PVS segmentation tool (22). Areas of WM hyperintensity (WMH) were segmented on the same images using the artificial intelligence–enhanced Lesion Segmentation Toolbox and the USCLobes Atlas. Whole-brain WMH volumes were corrected for total intracranial volume and log-transformed.

### [^18^F]PI-2620 PET/MRI

Radiosynthesis of [^18^F]PI-2620 was performed as described previously (2). Dynamic PET data (0–60 min postinjection; mean dose, 183 ±1.8 MBq) were acquired simultaneously with MRI on a Biograph mMR scanner (Siemens Healthineers). Details are provided in the supplemental materials and Supplemental Figure 2. Briefly, PET images were motion-corrected, coregistered to individual MPRAGE, and quantified using the multilinear reference tissue model 2 (23,24) in Qmodeling (25) to derive distribution volume ratio (DVRs) and relative perfusion (R1-derived) maps. The inferior cerebellum served as the reference region. Mean DVR was extracted from an a priori AD-signature meta–region of interest (ROI) covering the temporal lobe (26); global and temporal WM DVRs were also quantified. In addition, SUVR images (30–60 min postinjection) were generated. Hippocampal perfusion was estimated using R1 maps.

### Statistical Analyses

#### ROI-Based Correlational, Regression, and Mediation Models

Statistical analyses were performed in MATLAB (2024b); mediation analyses were conducted in *R* ( 2024.09.1 + 394). Continuous variables were standardized using *z*-scores. Linear models were used to examine effects of age group, age (within older adults), sex, and APOE4 status on tau PET DVRs. Associations between plasma biomarkers and tau PET DVRs were tested, adjusting for age and sex, with and without temporal WM DVR. Associations between tau PET DVRs and cognitive factor scores controlled for education and time interval between assessments.

To identify markers of age-related brain pathology explaining episodic memory performance, we tested whether candidate variables mediated age effects or explained variance beyond age. Predictors included meta-ROI DVR, total WMH volume, PVS burden, MTL thickness, hippocampal volume, hippocampal R1-derived relative perfusion, plasma AT term, and GFAP. Single-mediator causal mediation analyses (5000 Monte Carlo simulations) were performed using the mediation package in R, adjusting for sex and education; time-interval effects were retained when significant. Meta-ROI DVR was residualized for temporal WM DVR. Finally, significant predictors and demographic covariates were entered into a multiple regression model to estimate the total variance explained in memory performance.

#### Voxelwise Analyses of Tau PET

Additional voxelwise analyses (2-sample *t*-test, multiple regression) examined the spatial pattern of tau tracer uptake with regard to age, sex, APOE4 status, and plasma AD biomarkers in the whole brain. DVR images were warped to Montreal Neurological Institute (MNI) space using SPM12 (Statistical Parametric Mapping) and smoothed with a 6-mm fullwidth at half maximum gaussian kernel. Second-level analyses were performed using explicit (gray matter [GM] and WM) and implicit masking. Depending on the analysis, age, sex, and global WM DVR were included as covariates. One older participant had a meningioma in the posterior medial cortex and was excluded from all voxelwise analyses.

## RESULTS

### Sample Characteristics

Tau PET data were analyzed for 166 older adults (61–88 y; 44% females) after exclusions and 13 young adults (22–34 y; 38% females). Participant characteristics are summarized in Table 1, and participant selection is detailed in Supplemental Figure 1. All but 1 participant completed PET imaging within 2 y of cognitive testing (90% within 1 y); this individual was excluded from cognition-related analyses.

**TABLE 1.** Participant Characteristics

Variable	*n*	Value
Age at PET (y)	166	72.0 **±** 7.0 (61.1–87.8)
Sex	166	
Female	73	
Male	93	
Education (y)	166	15.1 **±** 2.2 (11–20)
*APOE4*	160	
Carrier	37	
Noncarrier	123	
*APOE2*	160	
Carrier	27	
Noncarrier	133	
Aβ_1–42_/Aβ_1–40_	159	0.087 **±** 0.012 (0.036–0.110)
p-tau_217_ (pg/mL)	159	0.126 **±** 0.085 (0.052–0.678)
p-tau_217_/Aβ_1–42_	159	0.00463 **±** 0.00321 (0.00163–0.02241)
GFAP (pg/mL)	159	58.4 **±** 24.1 (23.4–194.9)
Meta-ROI DVR	166	0.92 **±** 0.06 (0.78–1.09)
Time between PET and cognitive testing (y)	166	−0.50 **±** 0.35 (−2.24 to −0.07)
VLMT—delayed recall	166	10.2 **±** 3.0 (2–15)
RCFT—delayed recall	165	18.4 **±** 6.3 (3.5–33.5)
Mini-Mental State Examination	166	28.9 **±** 0.93 (25–30)

VLMT = Verbal Learning and Memory Test; RCFT = Rey-Osterrieth Complex Figure Test.

Data are expressed as mean ± SD, with range in parentheses. Five older participants carried both APOE4 and APOE2.

Mean tracer uptake patterns are shown in Figure 1. High binding was observed in the choroid plexus and substantia nigra, particularly in older adults (Fig. 1A), as well as in the meninges and soft tissue. WM binding was also evident, especially in young adults (Fig. 1B), and showed substantial interindividual variability. Visual rating results of WM binding are provided in Supplemental Figure 3.

**FIGURE 1.**
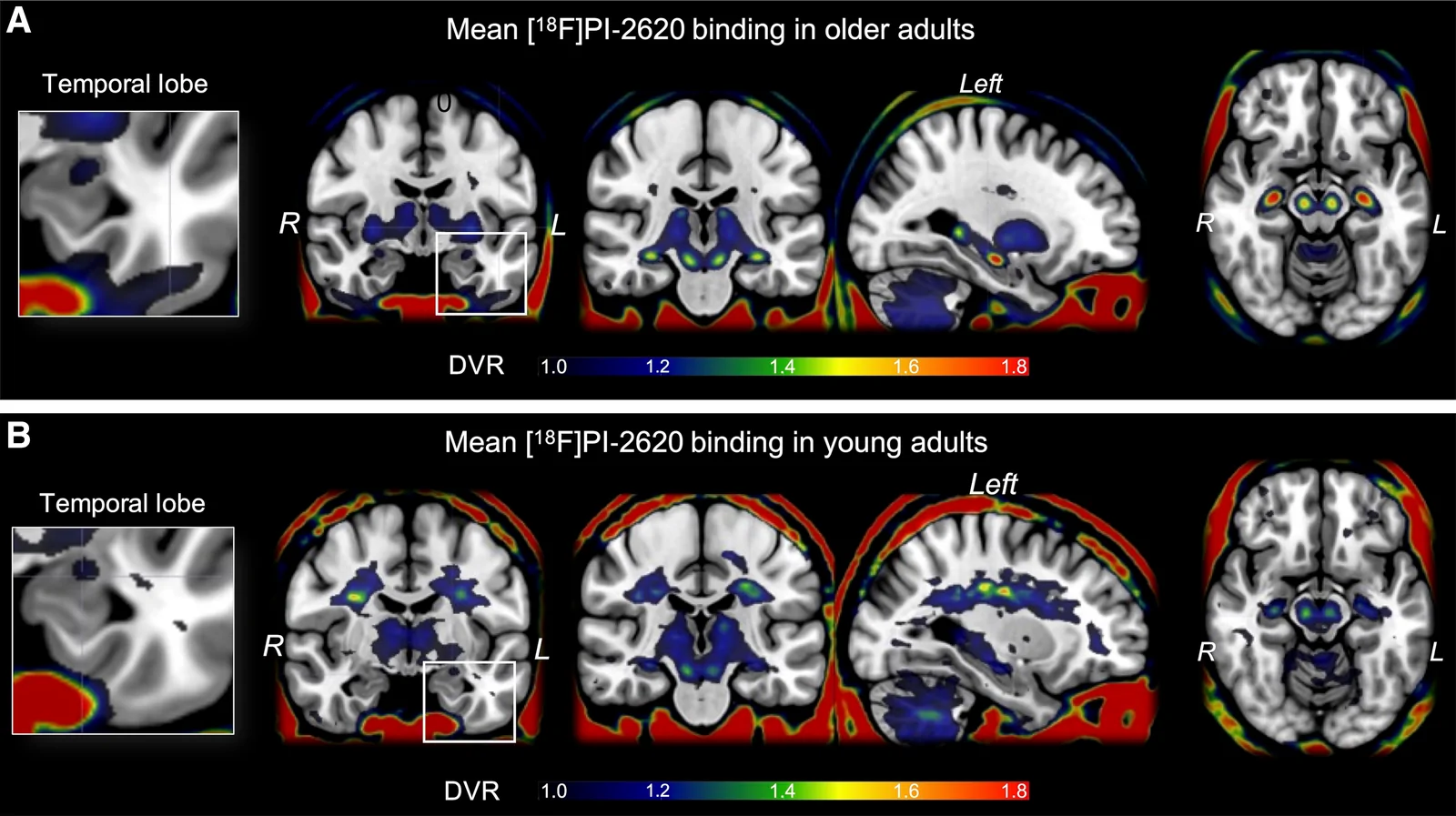
[^18^F]PI-2620 average binding potential maps. Mean tracer binding (DVR) image from 165 older adults (A) and 13 young adults (B). Images are scaled from 1 to 1.8 for display purposes, but DVR values of less than 1 were frequently observed. Supplemental Figure 4 shows DVR in meta-ROI.

### Associations of [^18^F]PI-2620 Binding with Sex, Age, and APOE4 Genotype

#### ROI-Based Analyses

Meta-ROI DVR was higher in older compared with young adults after adjusting for sex (β = 0.570, *t*(176) = 2.06, *P* = 0.041; Fig. 2A), with no significant difference for WM DVR (β = 0.032, *t*(176) = 1.55, *P* = 0.124). Within older adults, meta-ROI DVR was higher in females than males (β = 0.543, *t*(156) = 3.53, *P* < 0.001; Fig. 2B) and APOE4 carriers compared with noncarriers (β = 0.395, *t*(156) = 2.19, *P* = 0.030; Fig. 2C). Age was not associated with meta-ROI DVR (*P* = 0.80; Fig. 2D) but showed a positive association after adjusting for temporal WM DVR (β = 0.017, *t*(155) = 2.57, *P* = 0.011), potentially spilling into the GM. Global WM [^18^F]PI-2620 binding was weakly negatively associated with age (β = −0.026, *t*(156) = −2.27, *P* = 0.025; Fig. 2E) but not sex or APOE4 status within older adults (all *P* > 0.14). We explored the associations of WM DVR with scan timing, dose, body mass index, and WMH volume (adjusted for age and sex). None were significant (all *P* > 0.12), except for a trend-level negative association with body mass index (β = −0.042, *P* = 0.092). Similar associations were observed when considering meta-ROI SUVR and WM SUVR (results not shown). The association between DVR and SUVR values is shown in Supplemental Figure 4.

**FIGURE 2.**
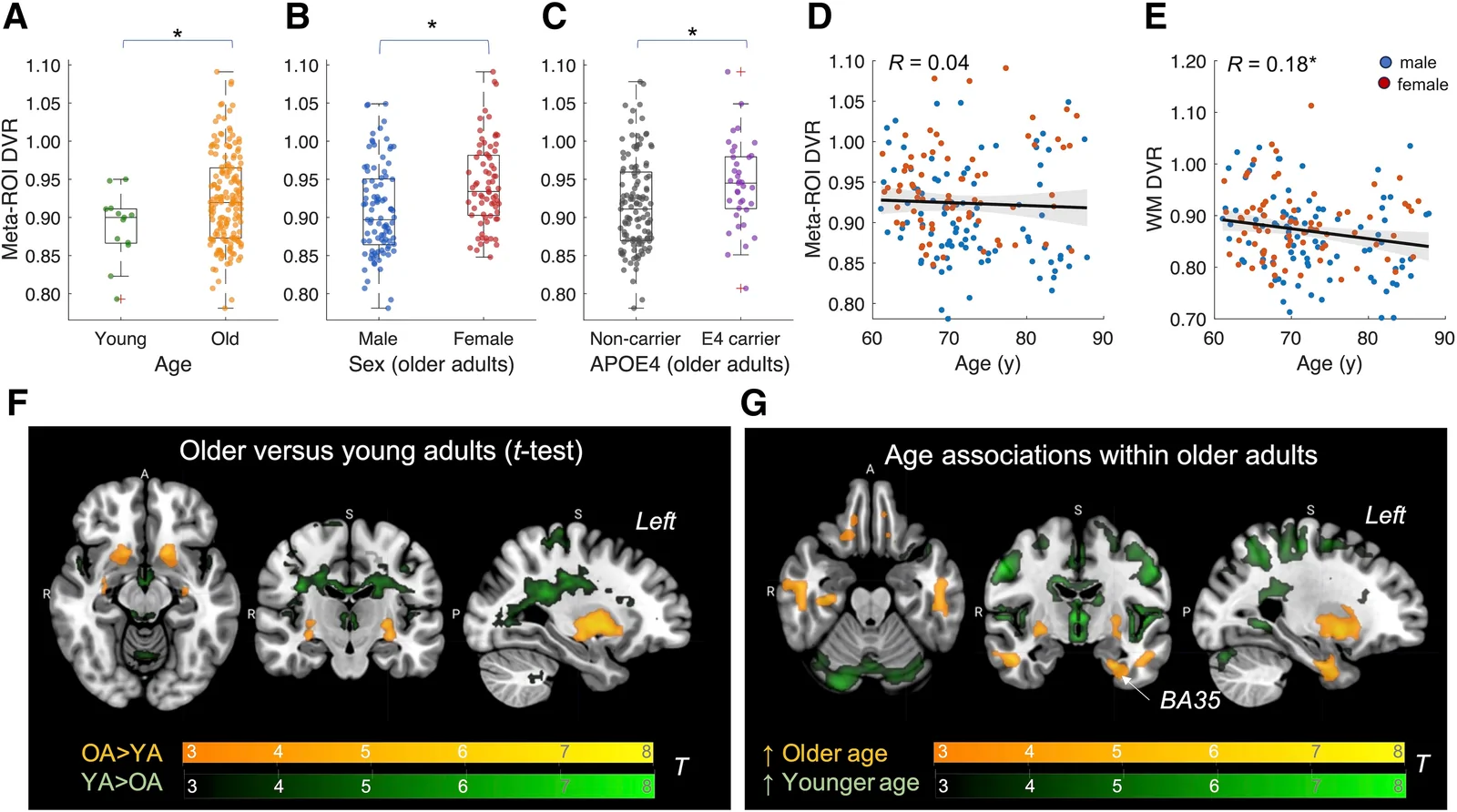
Associations of age, sex, and APOE4 genotype with [^18^F]PI-2620 binding. (A–E) ROI-based associations. (F and G) Voxelwise associations in MNI space depicted at cluster-level familywise error-corrected *P* of <0.05 and voxel-level *P* of <0.001. *Denotes significant group differences at *P* < 0.05; OA = older age; YA = younger age.

#### Voxelwise Analyses in MNI Space

Two-sample *t*-tests showed higher WM tracer binding (including, for example, corpus callosum and posterior periventricular WM) in young adults and higher basal ganglia binding in older adults (Fig. 2F), noting the small and unequal group sizes. Multiple regression analyses within older adults (sex-adjusted) revealed higher WM binding with younger age (Fig. 2G), with a similar pattern seen for the differences among age groups (Fig. 2F). While adjusting for global WM DVR, older age was related to elevated tracer binding in the anterior, medial, and inferior temporal lobes, as expected, as well as in the basal ganglia (Fig. 2G). Older females showed higher tracer binding than males across cortical GM, WM, and the outer cortical border (Supplemental Fig. 5), whereas no APOE4-related effects survived cluster-level correction.

### Relationship Between Tau PET and Plasma Biomarkers

#### ROI-Based Analyses

In regression models adjusting for age and sex, higher meta-ROI DVR was associated with higher p-tau_217_ (β = 0.22, *t*(155) = 2.70, *P* = 0.008) and AT term (β = 0.21, *t*(155) = 2.52, *P* = 0.013; Figs. 3A and 3B). Aβ_1–40_/Aβ_1–42_ and GFAP were not significantly related to meta-ROI DVR (all *P* > 0.5) (Fig. 3C; Supplemental Fig. 6). An overview of all associations among meta-ROI DVR, WM DVR, and the various imaging and plasma markers is provided in Supplemental Figure 7.

**FIGURE 3.**
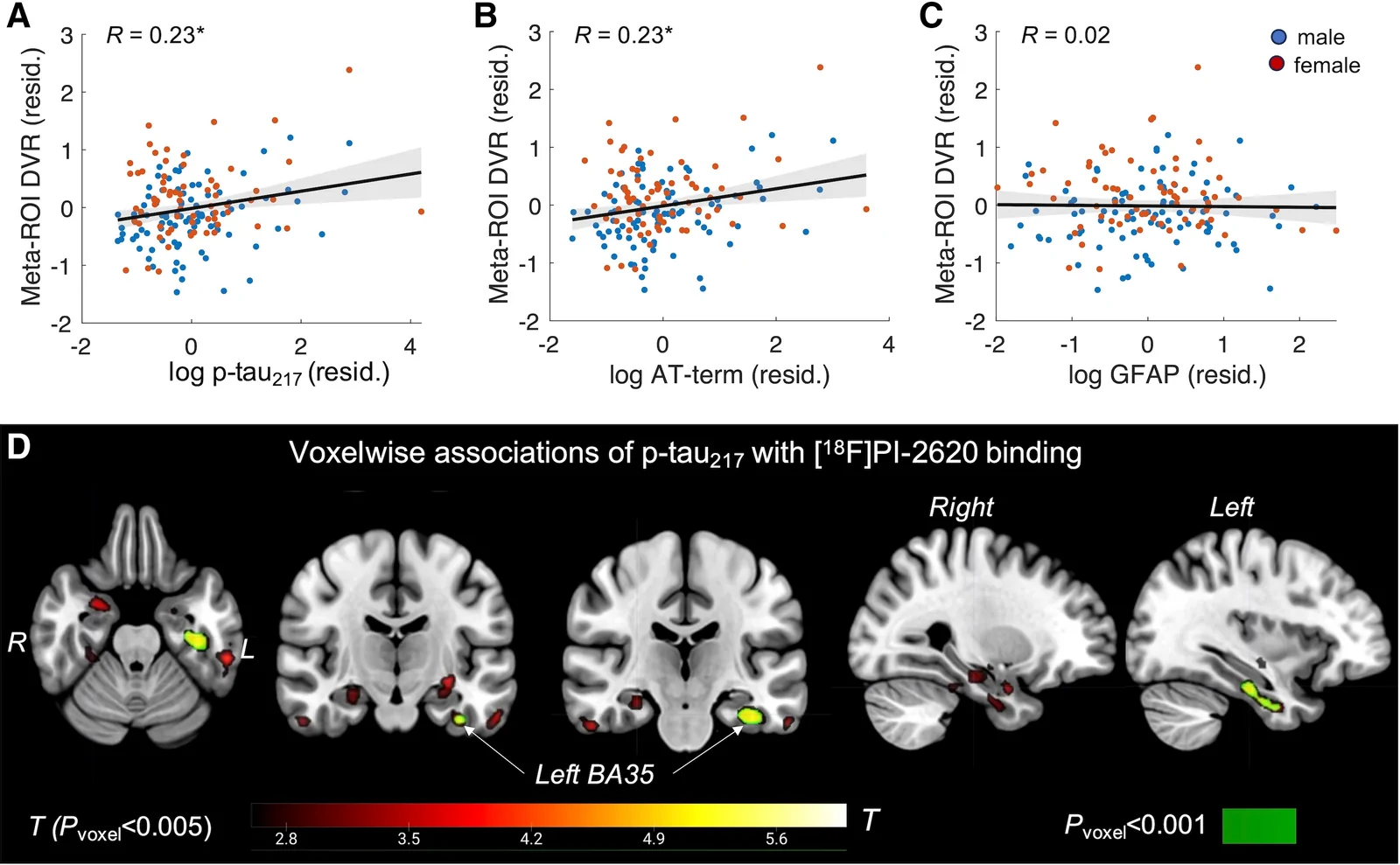
Associations of AD-related plasma biomarkers with [^18^F]PI-2620 binding. (A–C) ROI-based associations. Regression lines are plotted using standardized residuals after adjusting for age, sex, and WM DVR (latter for PET variables only). (D) Positive voxelwise associations depicted at cluster-level familywise error-corrected *P* of <0.05 and voxel-level *P* of <0.005 (hot color; liberal threshold) and voxel-level *P* of <0.001 (green). *P < 0.05; resid. = residual.

#### Voxelwise Analyses

Multiple regression analyses in MNI space showed that higher plasma p-tau_217_ was associated with elevated tau tracer uptake in the temporal lobe, hippocampus, and amygdala (cluster-level familywise error-corrected *P* < 0.05, voxel-level *P* < 0.005) (Fig. 3D). The association was strongest in left Brodman areas 35 and 36 (BA35/BA36) (voxel-level *P* < 0.001).

### Exploratory Factor Analysis of Latent Cognitive Components

Exploratory factor analysis identified 4 latent cognitive domains—semantic retrieval, visuomotor processing, phonemic fluency, and episodic memory—on the basis of factor loadings (Fig. 4A; Supplemental Results). Together, these factors explained 41% of the variance. Factors were estimated using oblimin rotation, revealing moderate interfactor correlations (Fig. 4B). Associations of age, sex, and education with domain-specific factor scores are reported in Supplemental Table 1.

**FIGURE 4.**
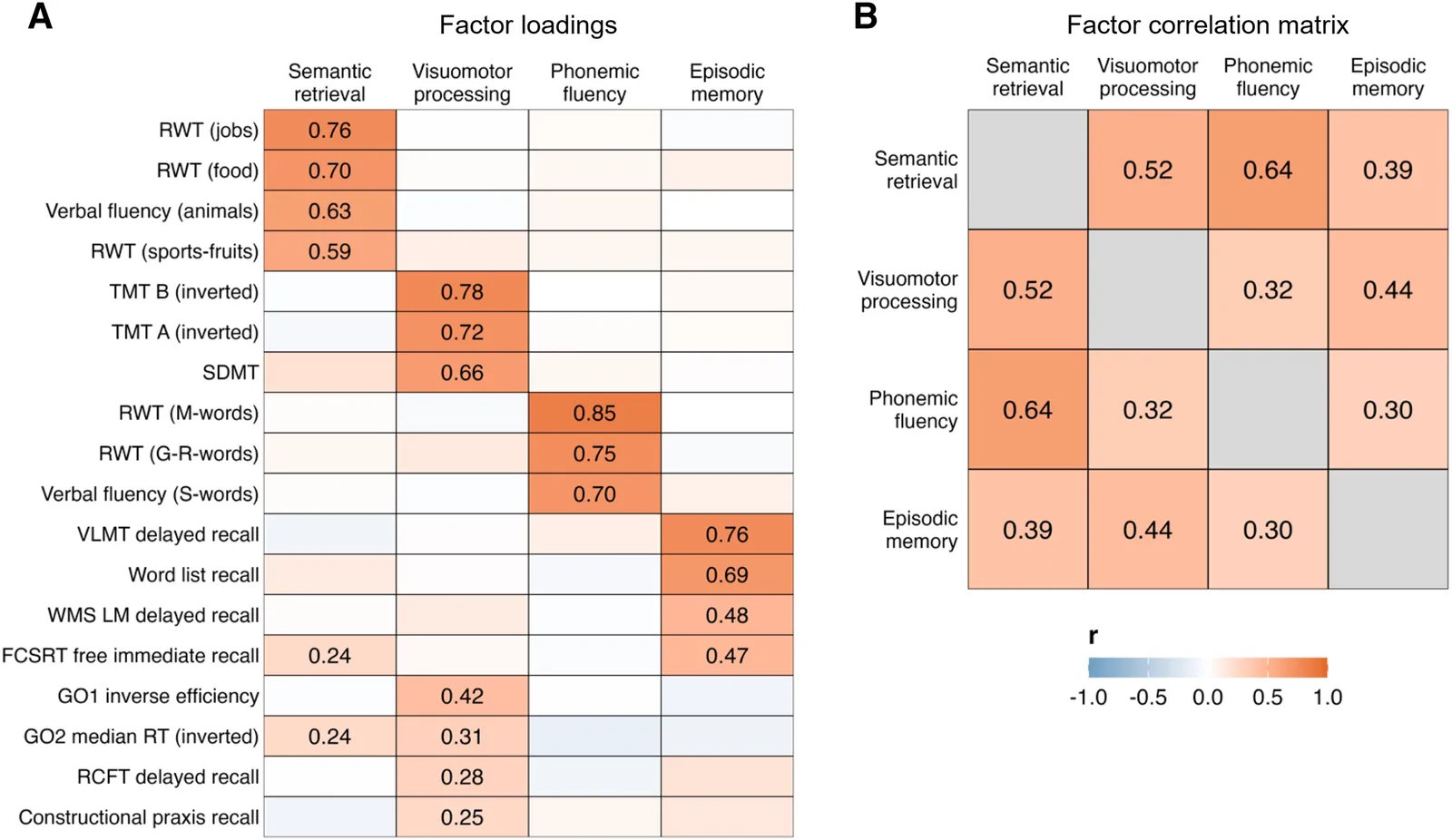
Exploratory factor analysis of cognitive components. Loadings of individual cognitive test scales of 4 identified cognitive factors (A) and interfactor correlation matrix (B). Factor loadings with |*r*| < 0.2 were not displayed. FCSRT = Free and Cued Selective Reminding Test; GO1 = first and GO2 second test of Go/No-go task; RCFT = Rey-Osterrieth Complex Figure Test; RT = reaction time; RWT = Regensburger Wortflüssigkeits-Test; SDMT = Symbol Digit Modality Test; TMT =Trail Making Test; VLMT = Verbal Learning and Memory Test; WMS LM = Wechsler Memory Scale Logical Memory.

### Relationship Between Temporal Lobe Tau PET Burden and Cognition

Higher meta-ROI DVR was related to lower episodic memory (β = −0.35, SE = 0.13, *t*(157) = −2.81, *P* = 0.006, false discovery rate–corrected *P* = 0.024; Fig. 5A), with no associations found with other cognitive domains. The negative association between meta-ROI DVR and episodic memory remained consistent without adjustment for temporal WM DVR and after adjusting for the plasma Aβ ratio (Supplemental Results).

**FIGURE 5.**
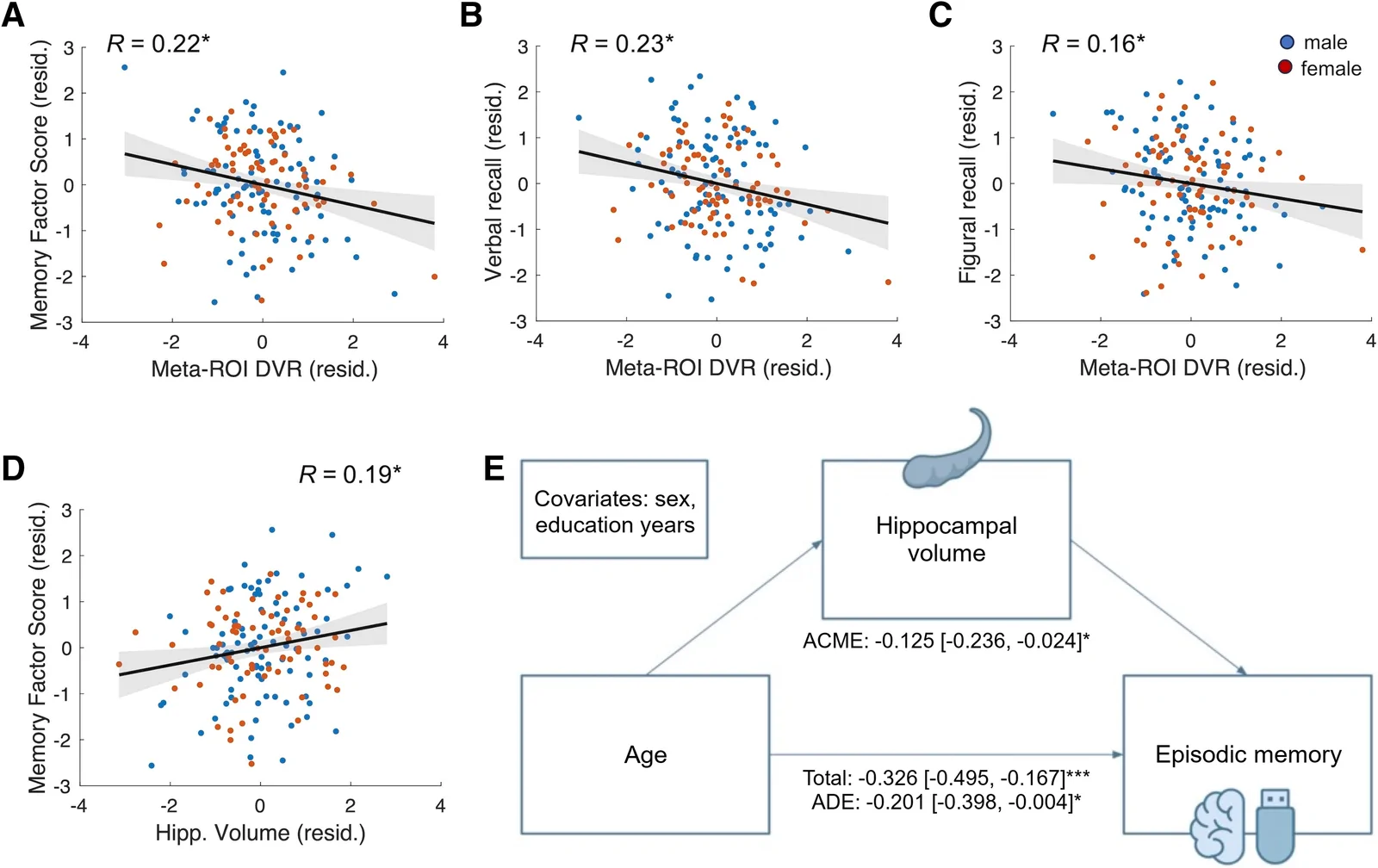
Associations of temporal lobe [^18^F]PI-2620 binding and hippocampal volume with memory. (A–C) Regression plots of meta-ROI DVR (residualized for age, sex, and temporal WM binding) versus memory factor score, VLMT delayed recall, and RCFT delayed recall (residualized for age, sex and education years). (D) Regression plot of bilateral mean hippocampal volume (residualized for age, sex, and total intracranial volume) and memory factor score (residualized for age, sex and education years). (E) Causal mediation model demonstrating significant partial mediation effect. *P < 0.05; ***P < 0.001; ACME = average causal mediation effect; ADE = average direct effect (after accounting for mediation effect); hipp. = hippocampal; resid. = residual; RCFT = Rey-Osterrieth Complex Figure Test; VLMT = Verbal Learning and Memory Test.

Supplemental analyses on test-specific associations of tau PET burden and delayed recall across the 4 performed memory tests revealed that higher meta-ROI DVR was related to worse delayed verbal and figural recall performance (all *P* < 0.03) (Figs. 5B and 5C; Supplemental Results).

### Multivariate Predictors of Episodic Memory

Next, we examined which brain and plasma markers explained poorer episodic memory and whether they mediated age effects or accounted for variance beyond age.

As expected, all pathology-related markers increased with age (Supplemental Fig. 8A), with the strongest effects related to hippocampal volume. After adjusting for sex and education, several of these brain measures predicted memory performance, including meta-ROI DVR, WMH, MTL thickness, hippocampal volume, and GFAP (Supplemental Fig. 8A). Mediation analyses showed partial mediation of the age–memory association by hippocampal volume (β = −0.125; 95% CI −0.236 to −0.024; *P* = 0.018) (Fig. 5E; Supplemental Table 2) as well as meta-ROI DVR, residualized for WM DVR (β = −0.044, 95% CI, −0.101 to −0.001; *P* = 0.042). However, the mediation was not significant for raw meta-ROI DVR (Supplemental Table 3) and thus should be considered with caution. Mediation models for age effects on semantic retrieval and visuomotor processing are shown in Supplemental Tables 3 and 4.

After adjusting for age, only hippocampal volume was significantly associated with memory (β = 0.20, SE = 0.08, *t*(159) = 2.43, *P* = 0.016; Supplemental Fig. 8B), aside from meta-ROI DVR (Fig. 5A). A multiple regression model explained 30% of the variance in memory performance (Supplemental Table 5), with meta-ROI DVR and hippocampal volume making independent contributions.

## DISCUSSION

In this study, we characterized binding patterns of [^18^F]PI-2620 across a wide age range of CU adults, including very old and young individuals. Tau tracer binding was higher in older than young adults and positively associated with female sex, APOE4 genotype, plasma p-tau_217_, and episodic memory deficits within older adults. We also identified off-target binding in several regions, including the choroid plexus, and variable WM signal that was more pronounced in younger participants.

Basal ganglia uptake increased with older age, a pattern also observed with other first- and second-generation tracers (13,23). For flortaucipir, age-related basal ganglia retention has been linked to regional iron content and monoamine oxidase, suggesting off-target binding (18,27). Regarding [^18^F]PI-2620, basal ganglia signal has been shown to reflect disease-specific target binding in 4-repeat tauopathies (2,28,29), which also correlates with iron deposition (30). These findings indicate that the age-related basal ganglia signal reflects a mixture of tau signal and off-target binding.

Age-related MTL binding within older adults emerged after WM adjustment, with peak effects seen in the anterior temporal cortex (BA35/BA36). Prior studies using different tau tracers have similarly reported age-related MTL binding in CU older adults, even in the absence of Aβ (31,32). WM binding of [^18^F]PI-2620 was highly variable (absent to focal), spatially irregular (unlike Aβ tracers), higher in younger participants, and unrelated to global WMH—arguing against an association with WM lesions or reduced uptake in lesions (33). A previous study likewise reported high WM SUVRs in both healthy control subjects and individuals with frontotemporal lobar degeneration (11), with no differences observed between diagnostic groups or demographic or acquisition effects. The WM effect is also unlikely to be driven by the kinetic modeling approach or the choice of reference region. We replicated the WM signal in a participant with particularly high binding using an alternative, unbiased image-derived input function pipeline using various reference regions (29,34) (results not shown). Although the biologic basis of this WM binding remains unclear, adjusting for temporal WM DVR, which might spill over into GM, slightly strengthened the expected associations between temporal lobe binding and age, plasma p-tau_217_, and memory. We also observed off-target binding in the meninges, skull, and choroid plexus, as previously reported in [^18^F]PI-2620 studies (7,11) and studies using first-generation tracers (9,18,35). This signal may spill over into adjacent regions (e.g., temporal cortex, hippocampus), particularly in the presence of atrophy, and should be considered when interpreting the results. Although bone is accounted for in advanced MRI-based attenuation correction, minor inaccuracies in regions adjacent to the skull cannot be fully excluded, which may also influence the extracerebral signal.

Our results of increased [^18^F]PI-2620 binding in women are consistent with previous reports for other tau tracers (36,37), even when accounting for Aβ. However, our whole-brain analysis revealed that women exhibited higher tracer binding at the GM–meningeal interface, consistent with previous observations that showed increased off-target retention in the meninges and skull across [^18^F]flortaucipir, [^18^F]RO948, and [^18^F]MK6240 (31,35,38), potentially related in part to lower bone density (39). Higher biologic vulnerability to AD pathology in women might be linked to hormonal decline during menopause, among other factors (40).

Our observation of elevated temporal [^18^F]PI-2620 binding in CU APOE4 carriers aligns with APOE4-associated MTL vulnerability seen with other tracers, independent of cortical Aβ (41), with APOE4 contributing to earlier tau PET positivity in CU individuals (32).

Temporal [^18^F]PI-2620 binding was further associated with higher plasma p-tau_217_ concentrations, but not Aβ ratio or GFAP. Voxelwise analyses indicated region-specific associations between p-tau_217_ and tau PET in the perirhinal/transentorhinal area, where tau tangles accumulate early (1). Recent head-to-head analyses in CU older adults showed moderate correlations between plasma p-tau_217_ and MTL tau PET SUVR (ρ = 0.43) and comparable performance in predicting cognitive decline (42). The absence of a relationship between [^18^F]PI-2620 and GFAP here is consistent with data indicating that plasma GFAP primarily reflects astrocytic responses to Aβ and has weaker or Aβ-dependent associations to tau PET (43).

Consistent with previous tau PET studies in CU cohorts (15–17), higher temporal lobe binding was specifically associated with poorer (verbal and figural) episodic memory performance, with a small-to-medium effect size independent of demographics. Hippocampal volume explained additional variance, and together these markers captured both age-related and age-independent variance in episodic memory. Larger-than-expected hippocampal volumes for a given age may further indicate brain reserve. Finally, in a model also including demographics, only about one third of the variance in episodic memory performance was explained, and none of the other markers (perfusion, PVS, GFAP, and WMH) contributed significantly. Similarly, a cross-sectional analysis including cerebrospinal fluid–based AD and inflammation biomarkers, WMH, and atrophy measures found independent associations of AD pathology (Aβ_42/40_) and hippocampal volume with memory, with all predictors explaining only 14% of the variance (19). In a multimodal analysis of 166 CU participants, latent factors reflecting vascular risk, WM injury, and perfusion predicted cognitive decline beyond Aβ and tau, and together with demographic and interaction terms explained approximately half of the variance in longitudinal decline (20).

These findings highlight the substantial proportion of unexplained interindividual variability in memory performance in old age beyond established markers of brain pathology. One possibility is that functional brain measures, such as synaptic activity or network connectivity, may capture reserve or compensatory processes or early dysfunction. However, studies in CU aging report only weak associations between functional connectivity and cognition (44), with most focusing on resting-state measures and rarely testing moderation of pathology–cognition relationships.

Modest effect sizes in CU cohorts likely reflect low pathologic burden, particularly in samples with strict exclusion criteria. Limited sensitivity of current methods to detect early tau pathology or neuronal dysfunction, together with variability in cognitive testing, may further contribute to unexplained variance. Thus, although tau pathology and hippocampal atrophy reliably relate to episodic memory, substantial heterogeneity remains.

This study had limitations, including restricted generalizability attributable to selection bias, limited sociodemographic diversity, and a cross-sectional design. Strengths include comprehensive phenotyping with detailed cognition, multimodal imaging, and plasma biomarkers, enabling integrative analyses. To our knowledge, this is the first study of tau PET to include young adults (recruitment is ongoing)—among whom tau accumulation is unlikely—although the small sample size warrants validation.

## CONCLUSION

Our findings suggest that [^18^F]PI-2620 is sensitive to MTL tau accumulation and memory deficits in aging, while highlighting the need to account for WM and off-target binding. Longitudinal studies incorporating additional neural markers are needed to explain interindividual variability in episodic memory and resilience to brain pathology.

## DISCLOSURE

Funding was provided by the German Research Foundation to Niklas Behrenbruch, Svenja Schwarck, Beate Schumann-Werner, Eóin Molloy, Constanze Seidenbecher, Björn Schott, Michael Kreissl, Emrah Düzel, and Anne Maass under Project ID 425899996 CRC1436 and to Anne Maass, Niklas Behrenbruch, Larissa Fischer, Constanze Seidenbecher, and Emrah Düzel under Project ID 362321501 RTG 2413. Computational PVS quantification was supported by The Galen and Hilary Weston Foundation under the Novel Biomarkers 2019 scheme (ref. UB190097) administered by the Weston Brain Institute and the Row Fogo Charitable Trust (ref. BRO-D. FID3668413). Henryk Barthel reports research support from Life Molecular Imaging (LMI); consulting or speaker honoraria from Hermes Medical Solutions, IBA, Lilly, Eisai, Novartis/AAA, and GE HealthCare; reader honoraria from LMI; dosing committee honoraria from Pharmtrace; and scientific advisory board honoraria from SOFIE Biosciences and Positrigo. Andrew Stephens is an employee of LMI. The Leipzig Nuclear Medicine group (Michael Rullmann, Andreas Schildan, Henryk Barthel, and Osama Sabri) holds a Chemical Manufacturing and Control contract with LMI related to the development of [^18^F]PI-2620. No other potential conflict of interest relevant to this article was reported.

## Notes

KEY POINTS **QUESTION:** How is [^18^F]PI-2620 binding related to age, sex, APOE4 genotype, plasma AD biomarkers, and memory function in CU older adults? **PERTINENT FINDINGS:** Dynamic [^18^F]PI-2620 binding was examined across a wide age range in CU adults. Higher temporal tracer binding was significantly associated with APOE4 genotype, female sex, and higher plasma p-tau_217_. Temporal tau burden and hippocampal volume independently predicted episodic memory and partially mediated age effects. Elevated WM binding was observed with younger age. **IMPLICATIONS FOR PATIENT CARE:** Our findings support the clinical utility of [^18^F]PI-2620 tau PET for early detection of tau pathology and for informing tau PET–based trial selection and stratification, while highlighting that variable WM binding—and potential spillover to GM regions—should be considered.
